# From Fetal to Neonatal Neuroimaging in TORCH Infections: A Pictorial Review

**DOI:** 10.3390/children9081210

**Published:** 2022-08-11

**Authors:** Giulia Lucignani, Alessia Guarnera, Maria Camilla Rossi-Espagnet, Giulia Moltoni, Amanda Antonelli, Lorenzo Figà Talamanca, Chiara Carducci, Francesca Ippolita Calo Carducci, Antonio Napolitano, Carlo Gandolfo, Francesca Campi, Cinzia Auriti, Cecilia Parazzini, Daniela Longo

**Affiliations:** 1 Neuroradiology Unit, Imaging Department, Bambino Gesù Children’s Hospital IRCCS, 00146 Rome, Italy; 2 Neuroradiology Unit, NESMOS Department, Sant’Andrea Hospital, La Sapienza University, 00146 Rome, Italy; 3 Department of Radiology, IRCCS Materno Infantile Burlo Garofolo, Via dell’Istria 65, 34137 Trieste, Italy; 4 Academic Department of Paediatrics, Bambino Gesù Children’s Hospital IRCCS, 00146 Rome, Italy; 5 Medical Physics Unit, Risk Management Enterprise, Bambino Gesù Children’s Hospital IRCCS, 00146 Rome, Italy; 6 Neonatal Intensive Care Unit, Department of Medical and Surgical Neonatology, Bambino Gesù Children’s Hospital IRCCS, 00146 Rome, Italy; 7 Department of Pediatric Radiology and Neuroradiology, V. Buzzi Children’s Hospital, Via Castelvetro 32, 20154 Milan, Italy

**Keywords:** congenital infection, TORCH, MRI, CMV, fetal imaging, neonatal imaging, SARS-CoV-2

## Abstract

Congenital infections represent a challenging and varied clinical scenario in which the brain is frequently involved. Therefore, fetal and neonatal neuro-imaging plays a pivotal role in reaching an accurate diagnosis and in predicting the clinical outcome. Congenital brain infections are characterized by various clinical manifestations, ranging from nearly asymptomatic diseases to syndromic disorders, often associated with severe neurological symptoms. Brain damage results from the complex interaction among the infectious agent, its specific cellular tropism, and the stage of development of the central nervous system at the time of the maternal infection. Therefore, neuroradiological findings vary widely and are the result of complex events. An early detection is essential to establishing a proper diagnosis and prognosis, and to guarantee an optimal and prompt therapeutic perinatal management. Recently, emerging infective agents (i.e., Zika virus and SARS-CoV2) have been related to possible pre- and perinatal brain damage, thus expanding the spectrum of congenital brain infections. The purpose of this pictorial review is to provide an overview of the current knowledge on fetal and neonatal brain neuroimaging patterns in congenital brain infections used in clinical practice.

## 1. Introduction

Congenital infections of the central nervous system (CNS) are peculiar pathologies of fetal and neonatal immature immune systems and developing brain structures.

According to Baldwin and Whitley, three criteria should be met to consider an infection as congenital: (1) the onset of symptoms within 48 h of birth, (2) serological confirmation, and (3) the exclusion of other pathologies [1].

In early gestational age, congenital infections may cause miscarriage, premature birth, and intrauterine growth restriction (IUGR). Alternatively or in association with these events, they can cause various abnormalities of the CNS, including microcephaly, parenchymal calcifications, white matter abnormalities, hydrocephalus, and cortical developmental disorder, with severe neurological outcomes [2]. On the other hand, late infections, occurring in the second and third trimester, may determine alterations of the white matter myelination and usually result in milder outcomes [3] (Table 1).

The acronym TORCH stands for Toxoplasma Gondii, Other Agents (i.e., Syphilis, Listeria and parvovirus B19), Rubella, Cytomegalovirus (CMV), and Herpes Virus [2], and identifies a group of pathogens transmitted from mother to child through the placenta or during the perinatal period through delivery or immediately after the birth [4].

Pathogens of the TORCH group present a strong CNS tropism due to the high metabolic activity of neuronal and glial cells during perinatal life, and may carry dire long-term sequelae both in asymptomatic and symptomatic newborns [5].

Fetal and neonatal imaging plays an important role in reaching a proper diagnosis and reflects CNS damage depending on gestational age at the time of infection [6].

Pre-natal and post-natal cerebral ultrasound (cUS) can detect cerebral calcifications and possible macroscopic anomalies, as it is the primary diagnostic tool when a TORCH infection is suspected in utero or postnatally. Although cUS remains the primary screening technique, it presents many limitations, since subtle cerebral parenchymal changes might not be detected. Therefore, fetal and neonatal brain magnetic resonance (MR) is crucial in the diagnostic assessment of suspected congenital infection [7,8]. Brain MR, performed during prenatal life or postnatally, can better identify malformations of cortical development, white matter damage, callosal dysgenesis, posterior cranial fossa abnormalities, ischemic-hemorrhagic lesions, and intraventricular synechiae [9].

Postnatally, and in addition to cUS, MR is useful for the early identification of the infection and for the follow-up. Although MR findings often overlap in different congenital diseases, MR is generally useful in identifying typical and atypical findings for a differential diagnosis between infectious and non-infectious disorders (i.e., PSEUDO-TORCH and inborn error of metabolism) [10].

Thus, the main radiological challenge in the perinatal evaluation of TORCH infections is to quickly reach the diagnosis, to formulate the correct prognosis, and to establish an optimal and early therapy.

The purpose of our review is to provide a neuroimaging overview of the present knowledge regarding pre- and peri-natal brain involvement in TORCH infections and in the emerging congenital infections (i.e., Zika virus and SARS-CoV2) with clinical and laboratory correlations.

## 2. TORCH

### 2.1. Cytomegalovirus Infection

Congenital cytomegalovirus (cCMV) infection is the most frequent congenital infection worldwide, with a birth prevalence rate ranging between 0.2 and 2% of live births [11].

CMV is a member of the herpes virus family. Following the primary infection, herpes viruses remains latent in cells with potential subsequent reactivation. Like other herpes viruses, CMV is neurotropic and replicates in the ependyma and germinal matrix. Moreover, a specific tropism for endothelial cells has been hypothesized. This may eventually lead both to the alterations of neuronal migration and to the occurrence of microcalcifications and periventricular cysts. It has been hypothesized that CMV brain damage is the result of ischemic events related to direct viral tropism of the capillary endothelium and secondary to thrombotic vasculitis [10].

Transmission to the fetus is possible through maternal infected body fluids (blood, urine, semen, cervical fluid, and breast milk) [10]. There are two types of possible maternal infections: primary infection, which occurs in the absence of pre-conception immunity; and non-primary infection, which occurs because of the reactivation of the virus in cells. The ability of pre-conception immunity to protect the fetus from symptomatic infection is still controversial. Nevertheless, the risk of long-term sequelae is comparable between the primary and non-primary infections in some prospective studies [12]. Furthermore, since the virus can reactivate from the latent state in the infected mother, maternal immunity does not necessarily protect the fetus from exposure.

Newborns affected by cCMV are classified as symptomatic in the presence of neurologic signs at birth (lethargy, seizures, difficult feeding), microcephaly (head circumference < two standard deviations below the mean for age and birth weight), chorioretinitis, hepatosplenomegaly, petechiae, elevated serum transaminase levels (>80 IU/L), cholestasis, thrombocytopenia (<100,000 platelets/mm^3^), and hearing impairment [13].

Approximately 10–20% of infants are symptomatic at birth and 40–60% of them will be at risk for long-term sequelae, including neurocognitive sequelae and hearing loss. However, 10–20% of initially asymptomatic children may also develop sequelae [14,15].

Prenatal diagnosis is made by serology tests such as IgG, IgM, and IgG avidity tests. Intrauterine infection may be confirmed by PCR detection of CMV DNA in the amniotic fluid (AF) after the 21st gestational week (GW) and at least six weeks after infection. At birth, infection may be confirmed by PCR assay in blood, urine, and saliva [16].

**Figure 1:**
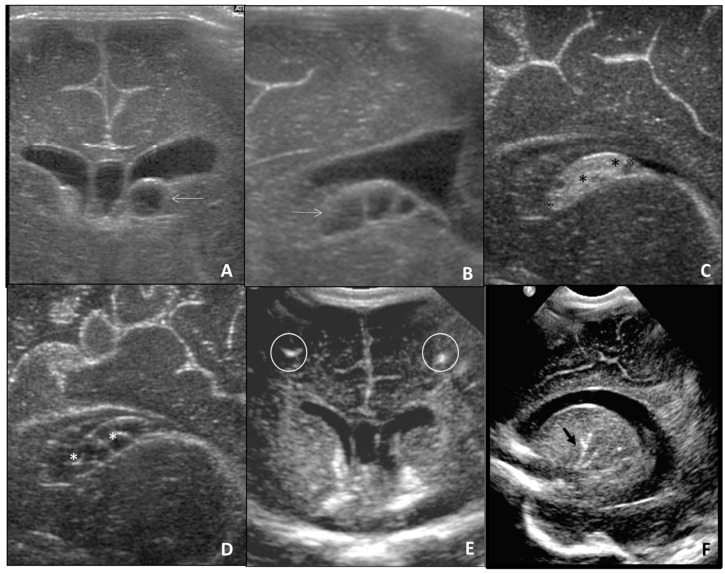
Cerebral US performed in neonate affected by CMV, showing periventricular cysts (dotted white arrows in (**A**,**B**)) and subependimal hemorrhage (black asterisks in (**C**)) evolving in periventricular cysts (white asterisks in (**D**). Cerebral US performed in neonate affected by CMV, showing frontal calcifications in the coronal plane (white circles in (**E**)) and calcifications of the lenticulo-striatal arterioles in the sagittal plane (black arrow in (**F**)).

The timing of infection is an important parameter to establish the severity of damage to the fetus, since infections contracted during the first trimester are more prone to cause serious neonatal and childhood diseases. In particular, the neuronal cell lines are formed between the 8th and the 20th weeks of gestation and neuronal migration occurs between the 24th and 26th weeks, with the highest proliferation of the germinal zone at the 26th week, while glial cells are formed at the end of neuronal proliferation. Therefore, an early infection will lead to a lack of proliferation of neurons and glial cells, while a late infection will lead to a reduction in neuronal cell organization with normal neuronal production and reduced production of glial cells [17]. Furthermore, direct and immune-mediated infiltration of the vascular endothelium has been described and may cause intraparenchymal and intraventricular hemorrhages, both in the pre- and the post-natal stage, and mostly in association with thrombocytopenia, liver failure, and immaturity of the CNS, mostly seen in premature children [18] (Figure 1).

**Figure 2:**
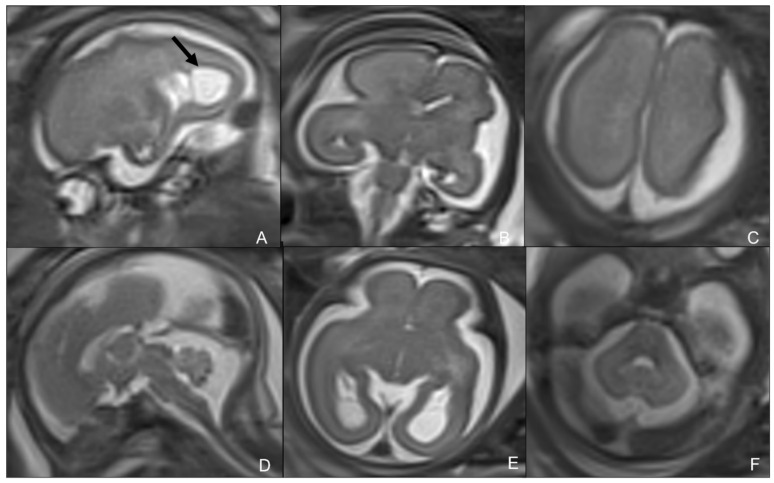
Fetus of 25 weeks of gestational age showing diffuse ventricular ectasis (**E**,**F**), cysternal and peri-encephalic spaces (**B**–**F**) coexisting with periventricular cysts (black arrow in (**A**)), and polymicrogyria (**B**,**C**). Courtesy of Mónica Rebollo Polo, Hospital Sant Joan de Déu, Barcelona, Spain.

During prenatal monitoring, fetal US can more frequently detect ventriculomegaly, microcephaly, periventricular calcifications, also subependymal cysts, intraventricular synechiae, white matter hyperechogenicity, callosal hypoplasia, lenticulostriate vasculopathy, sulcation and gyral abnormalities, and cisterna magna anomalies [19]. Post-natal cUS confirms the presence of the abovementioned cerebral alterations detected in prenatal cUS [20] (Figure 1 and Figure 2).

**Figure 3:**
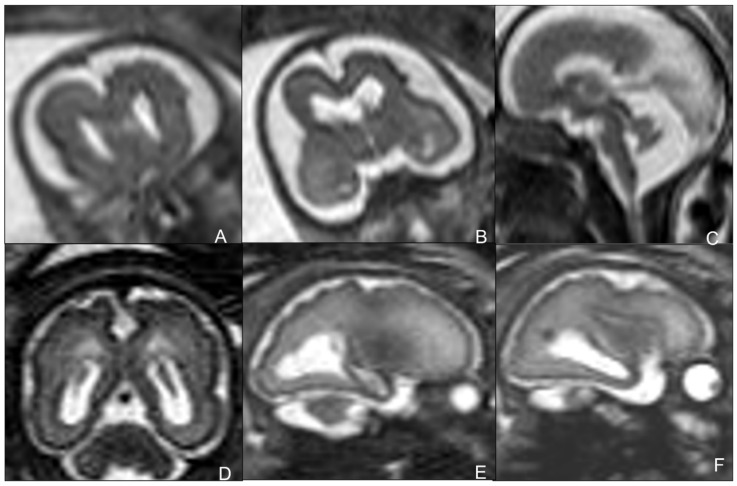
Fetuses of: 23 weeks of gestation age, showing polymicrogyria (**A**,**B**); 25 weeks of gestation age, showing cerebellar hypoplasia and polymicrogyria (**C**,**D**); 32 weeks of gestation age, showing white matter alterations (**E**,**F**).

**Figure 4:**
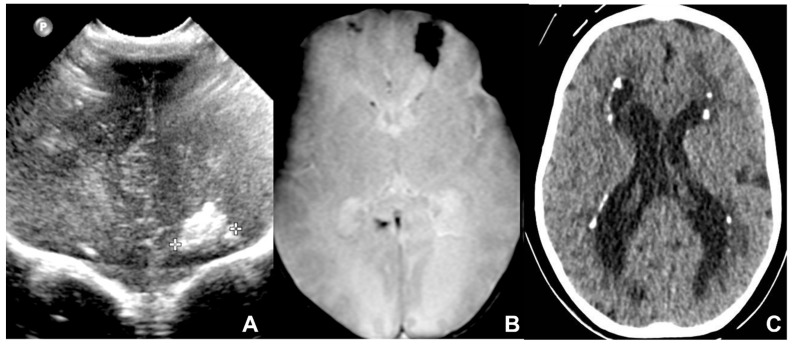
US (**A**) and MRI (**B**) performed in one-month-old neonate affected by CMV showing a coarse frontal calcification in the anterior coronal plane and on axial GE T2* sequence, respectively. Six-month-old child showing multiple and bilateral periventricular calcifications on axial CT (**C**).

MR is a second-level technique used in case of either negative or positive cUS, and is a problem solver in both pre-natal and post-natal contexts, thanks to its higher specificity and sensitivity in identifying typical CMV-related findings [21,22]. In particular, fetal MR is mandatory in the presence of at least one of the typical prenatal cCMV features at cUS. MRI common findings are T2-hyperintense lesions in the temporal poles with cystic-like appearance, cystic dilation of occipital horns, ventriculomegaly, white matter lesions (especially periventricular and sometimes with cyst-like appearance), and smudgy or thickened cortex on T2WI, better seen in the third trimester. Fetal MR may also rarely detect cerebellar hypoplasia. Typical intracranial calcifications are very difficult to detect at MR [19] (Figure 2 and Figure 3). The most frequent findings of post-natal brain MR are intracranial calcifications, which are more easily detectable by using gradient echo (GRE) or susceptibility-weighted imaging (SWI). Calcifications are also appreciable in CT scans, which should be used with caution due to the exposure of newborns to ionizing radiation [22] (Figure 4). Post-natal MRI is also able to better recognize cortical anomalies, such as lissencephaly, pachygyria, diffuse or focal polymicrogyria, and open or closed-lip schizencephaly. The first two forms are associated with a worse prognosis, and all are indicative of early congenital infection [10].

**Figure 5:**
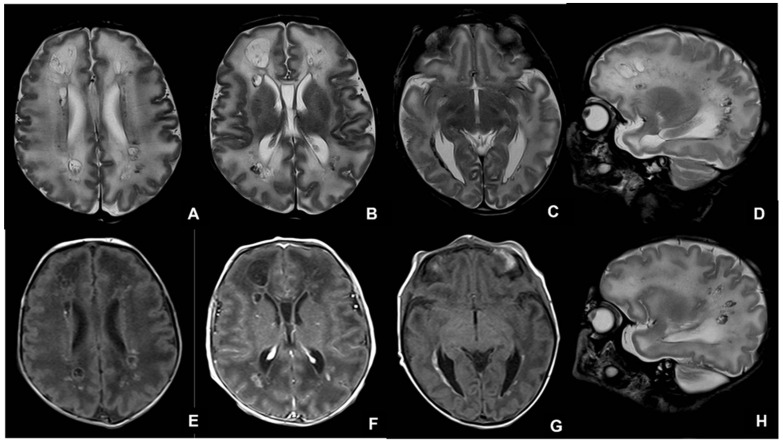
MRI performed in neonate affected by CMV in the first gestational trimester showing calcifications and cysts on axial (**A**–**C**) and sagittal (**D**,**H**) T2WI and on axial T1WI (**E**–**G**).

**Figure 6:**
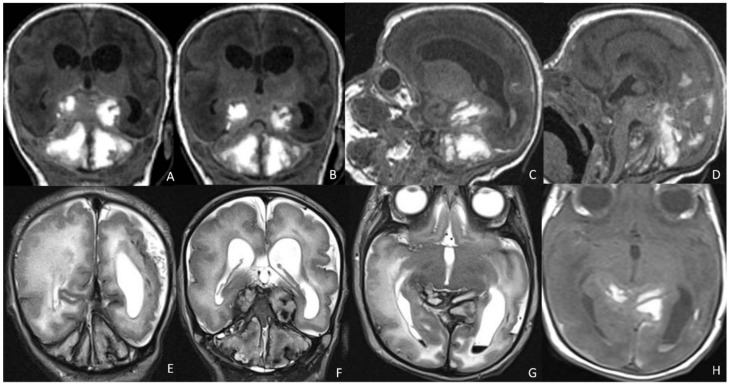
MRI performed in neonate affected by CMV in the first gestational trimester presenting with purpura, hepatosplenomegaly, coagulation, and neurological deficits, showing cerebellar and occipital hemorrhage coexisting with a subdural hematoma on coronal (**A**,**B**) and sagittal (**C**,**D**) T1WI and hemoventricle on coronal (**E**,**F**) and axial (**G**) T2WI and on axial T1WI (**H**).

**Figure 7:**
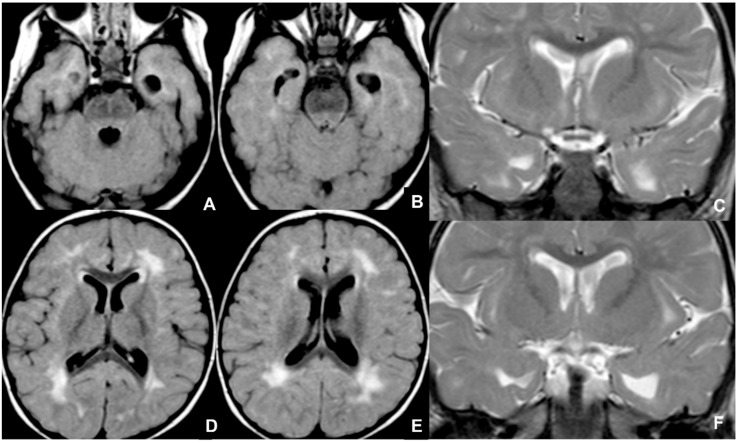
Seventeen-month-old child affected by CMV and presenting with post-natal deafness and neurocognitive retardation. MRI showed mesial temporal cysts on axial FLAIR (**A**,**B**), coronal T2WI images (**C**,**F**) and bilateral periventricular white matter alterations on axial FLAIR images (**D**,**E**).

Early post-natal MR is crucial to start a proper medical treatment to prevent sequelae. The presence of localizations of the infection in the central nervous system affects the duration of therapy [11] (Figure 5, Figure 6 and Figure 7).

Neuroimaging plays an important role in the definition of brain damage but can also give prognostic indications, since US and MR have high sensitivity and specificity for predicting neurological sequelae. In fact, there are many studies that have correlated the alterations identified by MRI with neurodevelopment in order to provide the clinician and families with more precise information to guide an early and correct treatment and follow-up [23]. Interestingly, Colonna et al. demonstrated that both asymptomatic and symptomatic newborns affected by cCMV develop long-term sequelae independent of the trimester of maternal infection [5]. Ultrasound is the most useful method in identifying infected newborns with major brain involvement, although not very reliable in predicting the long-term outcome, while MR correlates more precisely with a prognostic evaluation of neurological development [12]. Particularly, confirmation of cerebral involvement in deaf patients with sensorineural hear loss (SNHL), who could benefit from cochlear implants, is essential to know the presence of brain alterations, such as cortical malformations or white matter abnormalities, which could change implant efficacy [24,25].

A differential diagnosis includes genetic leukodystrophies such as leukoencephalopathy with subcortical temporal cysts, megalocephaly, and vanishing white matter disease [10].

Therapy during pregnancy consists of antiviral drugs (valaciclovir) to reduce the likelihood of fetal transmission or to ameliorate fetal sequelae. The administration of hyperimmune immunoglobulins has been debated and does not find uniform consensus. In neonates, therapy consists of antiviral drugs (ganciclovir/valganciclovir) [16].

### 2.2. Toxoplasmosis

Toxoplasmosis is the second most common congenital infection after CMV, with a prevalence ranging between 0.1 to 1 per 1000 live births in endemic countries [26]. Congenital toxoplasmosis is caused by intrauterine infection with the protozoan parasite Toxoplasma gondii. Pregnant women contract the disease by ingesting the oocysts of Toxoplasma gondii (T. gondii) nested in vegetables and raw meats, and infected domestic cats are a frequent potential vehicle of the pathology. The rate of transplacental transmission varies from 6% at 13 weeks of gestation to 72% at 36 weeks [27]. Despite that, nearly 80–90% of children are asymptomatic at birth, and disease severity is related to the date of maternal infection, the earlier being the more severe and frequently resulting in fetal death [28]. Clinical signs and symptoms may be appreciated at birth or appear several days to weeks later and may be related to nervous system involvement or be systemic. The most common are hydrocephalus, seizures, meningoencephalitis coexisting with chorioretinitis, and microphthalmos. In approximately 10% of patients, the triad of intracranial calcifications, chorioretinitis, and hydrocephalus is identified [29]. Generalized signs and symptoms are hepatosplenomegalies, anemia, and hyperbilirubinemia [30,31].

Prenatal diagnosis is based on maternal serology and amniotic fluid PCR analysis, while the detection of T. gondii specific IgM or IgA in infants’ serum guides postnatal diagnosis [31,32]. The persistence of IgG in patients at one year of age represents the gold standard for diagnosis of congenital infection [33].

**Figure 8:**
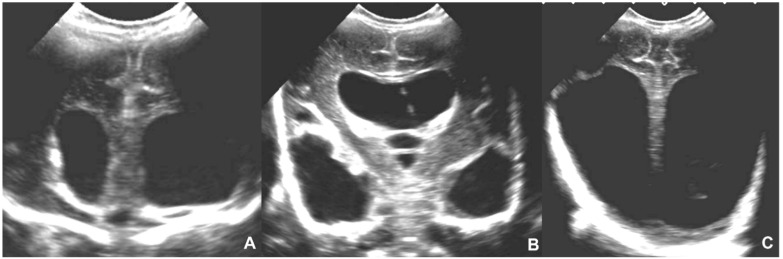
Four-day-old neonate affected by Toxoplasmosis and undergoing the cerebral US in the coronal plane, showing massive supratentorial ventricular dilatation (**A**–**C**) and linear subependymal calcifications (**A**,**B**).

**Figure 9:**
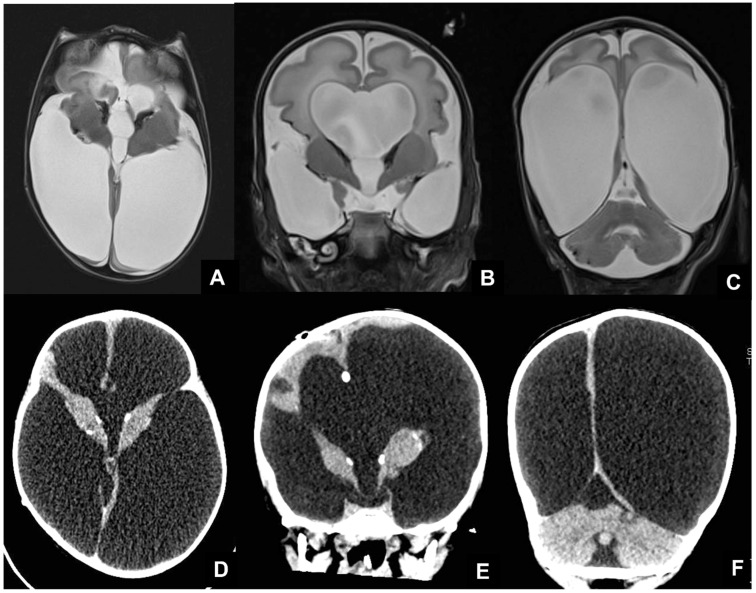
Pediatric patient affected by congenital CMV, undergoing MRI at four days of birth (**A**–**C**) and CT at six months of life (**D**–**F**), showing massive supratentorial ventricular dilatation and linear subependimal calcifications.

**Figure 10:**
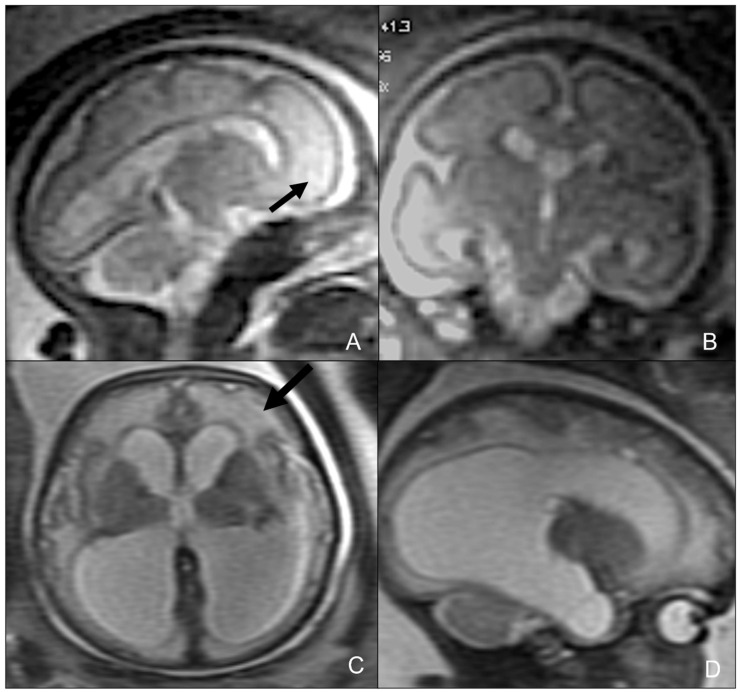
Fetus at 29 weeks gestational age affected by Toxoplasma, showing destruent frontal and insular white matter lesions (black arrow in (**A**,**B**)). Fetus of 34 weeks of gestation age affected by Toxoplasma, showing ventricular dilatation coexisting with destruent frontal white matter lesions (black arrow in (**C**,**D**)).

Radiological findings may range widely from minimal signs to extremely diffuse and severe abnormalities. Fetal MRI is a pivotal exam to identify Toxoplasmosis typical features and evaluate the severity of radiological findings. The most frequent findings are hydrocephalus, which is caused by aqueduct occlusion/stenosis secondary to epididymitis; ventricular dilatation, especially in the atria due to adjacent white matter involvement; and extensive cerebral calcification, which is more common in the basal ganglia, thalami, cerebral cortex, and periventricular tissue. Less common findings are microcephaly or macrocephaly, and porencephaly or hydranencephaly occurring in the second trimester [8,30,34]. At birth, cranial sonography is the first imaging performed and it is often followed by cross-sectional techniques such as CT, which better depicts cerebral calcifications, and MRI, showing similar radiological features as fetal MRI [35,36] (Figure 8, Figure 9 and Figure 10).

**Figure 11:**
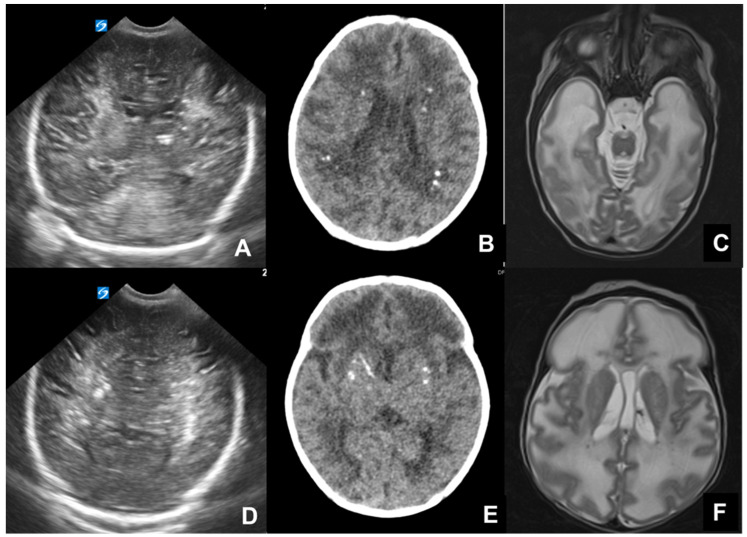
Twenty-day-old neonate affected by Aicardi-Goutieres type 1 syndromes, namely a pseudo-TORCH disease, showing white matter alterations, calcifications, and cystic vacuolization on US (**A**,**D**), CT (**B**,**E**), and MRI (**C**,**F**).

A differential diagnosis is made with CMV because its clinical and laboratory manifestations may resemble those secondary to T. gondii infection. In congenital toxoplasmosis, macrocephaly and chorioretinitis are more common, while in CMV cortical malformations such as polymicrogyria are more frequently found. Intracranial calcifications are shared by the two pathologies, but generally congenital toxoplasmosis shows a more peripheral distribution of calcifications compared to CMV [10]. Other differential diagnoses include TORCH and pseudo-TORCH pathologies and Aicardi-Goutières syndrome (Figure 11).

Toxoplasmosis prevention includes prenatal tests and preventive lifestyle habits such as avoiding raw meat and fish, peeling or washing fruits and vegetables, and cleaning surfaces and utensils used to prepare raw meat/poultry/fish and unwashed fruits and vegetables. In the case of domestic cats, pregnant women should avoid changing cat litter or do it with gloves and making sure to wash their hands afterward, avoid feeding cats raw meat, and keep cats inside to prevent infection [37]. Prophylaxis to prevent fetal infection is only active in parasitic forms and includes two different protocols: spiramycin, which is not efficient after 18–20 weeks of gestation due to incomplete passage through the placenta, and the combination of sulphonamides-pyrimethamine-folinic acid, which is efficient even in the latter part of pregnancy in case of fetal infection if the treatment is started within four weeks from maternal infection [38]. In case of fetal infection, early diagnosis and prolonged postnatal treatment improve children’s prognosis and consist of sulfadiazine—pyrimethamine and hydrocephalus shunting [39]. Recently, alternative medical protocols have been suggested. In particular, Buonsenso et al. demonstrated that the association of spyramicine-trimethoprim-sulfamethoxazole is not inferior to pyrimethamine-sulfadiazine and is more effective compared to spyramicine alone in reducing maternal-fetal transmission of Toxoplasmosis; and Valentini et. Al. demonstrated that the combination of spiramycin-cotrimoxazole has significant efficacy in reducing maternal-fetal transmission when compared to spyramicine and the combination pyrimethamine-sulfadiazine [40,41]. Sequelae may be limited with treatment and include seizure, spasticity, and mental retardation. Overall mortality is approximately 11–14% [29,42].

### 2.3. Rubella

The rubella vaccine and efficient immunization programs in developed countries lead to a massive decrease in congenital rubella syndrome (CRS), which still represents a major health issue in developing countries. Currently, nearly 100 cases of CRS occur worldwide. Rubella virus only affects humans, and transmission occurs through contaminated respiratory secretions postnatally. Vertical transmission during pregnancy occurs through the transplacental spread, and fetus sequelae are related to the timing of transmission. During the first trimester, nearly one-third of infections result in miscarriages. Infections contracted within the first 10 weeks of gestation present morbidity of about 90%, while at 16 weeks of gestation the morbidity is between 10 and 20%, with various sequelae such as deafness, blindness, motor and neurological defects, congenital heart disease, and mental impairment. Although clinical presentation may vary, the classic triad of CRS is represented by ocular anomalies, sensorineural deafness, and cardiac diseases. Morbidity is mild during late pregnancy, with congenital defects occurring rarely if transmission occurs after the 21st week of gestational age [43,44,45,46,47]. In childhood, rubella is common, presenting with fever and rash [48].

**Figure 12:**
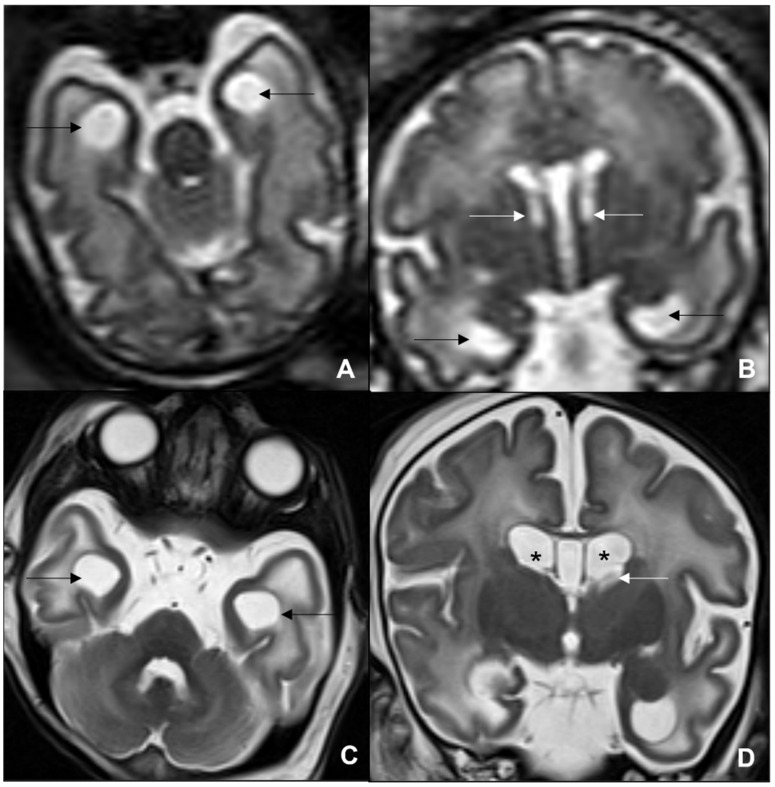
Fetal MRI performed on a fetus affected by Rubella at 27 weeks of gestational age, showing bilateral temporal cysts (black arrows in (**A**,**B**)), and subependymal cysts (white arrows in (**B**)). Neonatal MRI performed on the same patient at six days after birth, showing temporal cysts (black arrows in (**C**,**D**)) ventricular dilatation (black asterisks in (**C**,**D**)), and subependymal cysts (white arrow in (**D**)).

During the first trimester of gestation, serologic screening for rubella IgG antibodies is available, even if positive serum rubella IgM antibodies represent the most accurate biomarkers for diagnosis of acute infection [43,44,45,46,47]. To further confirm the diagnosis and exclude IgM false-positive results, an IgG avidity test may be performed to confirm that the infection is recent [49]. Fetal US and MR are excellent techniques to evaluate fetuses and neonates affected by CRS. The fetal US shows ventriculomegaly, ependymal irregularity and subependymal cysts, lenticulostriate vasculopathy, and diffuse calcifications. Fetal MR and neonatal MR may show microcephaly, ventriculomegaly, myelination delay, and extensive multifocal white matter (WM) hyperintensity, which appears to be predominantly frontal but rarely temporal, in contrast to CMV infection. Associated features are cerebellar hypoplasia and polymicrogyria. CT use is limited because of radiation exposure and typically shows ventriculomegaly, multifocal regions of WM hypodensity coexisting with periventricular, and basal ganglia calcifications and cysts [8,50,51,52,53]. Progressive rubella panencephalitis (PRP) is a late and rare presentation of CRS with an incidence of 1 in 6000 cases of rubella infection. PRP represents a rare neurodegenerative disorder that causes diffuse WM destruction, perivascular inflammation, gliosis, and cerebellar atrophy. MR may better depict demyelinating lesions and gliosis leading to diffuse atrophy, while CT shows diffuse periventricular calcifications (Figure 12) [53,54,55]. Differential diagnosis with other “TORCH” infections may be complex. Congenital rubella syndrome rarely presents with hepatosplenomegaly and jaundice compared to T. Gondii and CMV, while there is a higher rate of cataracts and congenital heart disease. Other common features are microcephaly, chorioretinitis, sensorineural hearing loss, and “blueberry muffin”, a sign of extramedullary hematopoiesis [8,43].

No specific therapy for Rubella infection is available. Therefore, therapy is tailored and related to the specific clinical presentation ranging from vision and sensorineural impairment to learning disabilities.

### 2.4. Herpes Simplex

Herpes simplex virus (HSV) is a rare cause of congenital infection transmitted from mother to child during pregnancy and more frequently during childbirth or in the peripartum period. Despite its low incidence, intrauterine HSV infection is important because it can have potentially catastrophic consequences such as death or severe neurodevelopmental disability [56]. The viral strains most frequently involved in the infection are HSV type 1 and type 2. The latter is responsible for maternal infection in the genital area, and it is the most common cause of congenital and neonatal infection, though in recent years there has been a gradual increase in neonatal HSV 1 infection, which is responsible for oral mucosa infections [57,58]. Transmission during pregnancy (congenital) occurs only in 5% of cases, through a placental passage or retrograde through broken or intact amniotic membranes. Late-onset infections are acquired during childbirth or peripartum and represent about 85% of cases [58,59]. Clinical manifestations of perinatal and neonatal HSV-1 and 2 infections are divided into three categories: SEM (skin, eye, and/or mouth), CNS disease, and disseminated disease. In the forms transmitted in utero, infants generally present with a combination of skin lesions, chorioretinitis, and microcephaly or anencephaly. Forms with predominant skin involvement show a better prognosis with a low risk of dissemination, while mucosal, ocular, or respiratory tract neonatal infections are at higher risk of dissemination both with and without CNS involvement and are associated with a worse prognosis. Thirty percent of neonates with HSV infection develop CNS involvement, especially if affected by HSV-2 infection. Clinical manifestations include seizures, cerebral palsy, neurodevelopment delay, and microcephaly [60]. HSV diagnosis is based on viral DNA isolation from biological fluids or in cerebrospinal fluid in case of CNS involvement [61]. 

**Figure 13:**
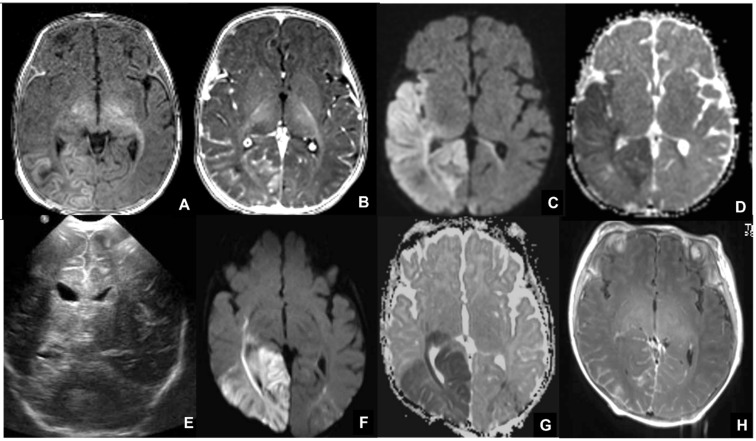
MRI of neonate affected by herpes virus type 1 (**A**–**D**) presenting with seizures and right hemiparesis, showing cortical-subcortical signal alteration on pre-contrast T1WI (**A**), contrast enhancement on post-contrast T1WI and diffusion restriction on DWI/ADC (**C**,**D**) in the right parietal-insular region. Neonate affected by herpes virus type 2 undergoing US, showing cortical-subcortical hyperechogenicity in the right posterior temporal and left parietal regions (**E**), and undergoing MRI, showing diffusion restriction on DWI/ADC (**F**,**G**) and contrast-enhancement in the abovementioned areas (**H**).

Neuroimaging in HSV infection is useful to suggest the diagnosis and prognosis but may be challenging because of potential overlap with the other congenital CNS infection imaging patterns. The fetal US shows different brain abnormalities, the most common being ventriculomegaly, agenesis of the corpus callosum, porencephaly, microcephaly, hydranenccephaly, and microphthalmia. Post-natal MR is the gold standard for the diagnosis thanks to the availability of both morphological and advanced functional sequences such as perfusion arterial spin labeling (ASL), and spectroscopic (MRS) sequences (Figure 13).

During the acute and subacute phases of infection, there is a prevalent involvement of the cortical grey matter and the subcortical white matter of the temporal-insular regions. There is usually the sparing of basal ganglia, capsules, brainstem, and cerebellum [62]. The rare involvement of basal ganglia and internal capsule is associated with a worse clinical outcome. In these cases, a differential diagnosis should be made with hypoxic-ischemic injury. However, HSV-related damage usually involves multiple brain regions with an asymmetric pattern that does not follow vascular territories. Moreover, DWI (diffusion-weighted image) sequences demonstrate initial cortical involvement followed by subcortical white matter involvement in the initial phase of the infection. Regions characterized by initial diffusion restriction undergo a gliotic and malacic transformation in the late phase of the infection [63]. Contrast agent administration may demonstrate mild meningeal enhancement in the involved regions [61]. Perfusion MR with the ASL technique may provide additional prognostic information compared to conventional imaging, such as regions of hypo-perfusion, which are associated with poor outcome. On the other hand, the presence of hyper-perfusion seems to be related to the development of seizures [64,65]. 

Acyclovir is the treatment of choice for neonatal HSV disease. In particular, it has been proved that a six-month-therapy with acyclovir results in an improved neurologic outcome [66].

### 2.5. Other and Emerging Viral Agents 

The TORCH “other” group of infections includes a miscellany of agents that can be vertically transmitted, such as Treponema pallidum, human immunodeficiency virus [HIV], parvovirus, Listeria monocytogenes, and varicella-zoster virus. These pathogens are related to a huge and non-specific spectrum of congenital neuronal injuries including meningitis, hydrocephalus, inflammatory vasculitis, sensorineural hearing loss from syphilis infection [67], meningitis and ventriculitis in Listeria infection [68], calcifications, brain atrophy and vasculitis in HIV infection [69], parenchymal calcifications, arterial infarction, cerebellar hemorrhage, and cortical malformations in parvovirus infection [70]. Recent literature underlined the importance of widening the spectrum of the “traditional TORCH” to include a variety of other congenital infectious etiologies, and proposed additional important direct diagnostic investigations, such as radiology, ophthalmology, audiology, microbiology, and PCR testing, to the serology-specific investigations included in the “TORCH screen” [71].

In the last decade in particular, two emerging viruses have gained attention: the Zika virus (ZIKV), after its outbreak in Brazil in 2015 [72], and Severe Acute Respiratory Syndrome Coronavirus-2 (SARS-CoV-2), which is the cause of the ongoing COVID-19 pandemic.

Zika virus is a single-strand RNA flavivirus, transmitted by infected female mosquitos, such as the Aedes mosquito [72]. The in utero infection potentially leads to adverse pregnancy and a variety of severe brain anomalies. Materno–fetal transmission can occur during any pregnancy period, though the earlier the infection the more severe the damage, with the greatest risk in the first-trimester infections. ZIKV is both neurotropic and glyotropic, preferentially targeting neural progenitor cells. Therefore, clue processes in brain development such as neuronal growth, proliferation, migration, and differentiation are disrupted, resulting in abnormal brain development [69].

The main clinical feature of congenital Zika infection is microcephaly, often associated with abnormal head shape with overriding sutures and redundant skin folders, probably because of the progressive development of bones and skin in comparison to the arrested brain development [7,72]. Other clinical findings are hypertonia and hyperreflexia, seizures, arthrogryposis, ocular abnormalities, and sensorineural hearing loss. Since the Zika infection in adults is often asymptomatic, prenatal diagnostic imaging is very important for screening pregnant women residing in or traveling to endemic areas. Prenatal cUS is considered as the first-line examination, eventually followed by fetal MR for a better depiction of brain abnormalities [69,70,71,72].

Newborns with suspected or confirmed diagnoses of congenital ZIKV infection should undergo MR as the imaging tool of choice to correctly define brain findings related to congenital ZIKV infection and to establish its severity. Microcephaly is the hallmark of the disease, especially if contracted in the first trimester, in which usually the brain is atrophic with ventriculomegaly and pseudocysts in the occipital horns. Almost all infants affected by congenital ZIKV infection present cerebral calcifications, which are characteristically located at the corticomedullary junction within the frontal and parietal lobes, though they can be present in the thalamus, basal ganglia, cortex, and periventricular regions as well. The peculiar distribution of brain calcifications is an important element to make a differential diagnosis between ZIKV and other congenital infections such as CMV and Toxoplasmosis. Other brain findings include malformations of cortical development such as polymicrogyria, gyral simplification, pachygyria-lissencephaly, and opercular dysplasia, while heterotopia is less common. Corpus callosum and posterior fossa abnormalities may be present. Abnormal white matter signal is present in the majority of infants, and it is likely caused by delayed myelination or dysmyelination. MR may also detect a thin spinal cord due to severe hypoplasia of the corticospinal tracts in addition to motor nerve cell degeneration and loss, gliosis, and small or coarse foci of calcification, with relative sparing of the sensory neurons. Spinal motor cell loss is thought to explain intrauterine akinesia, arthrogryposis, and neurogenic muscle atrophy [73]. Orbital abnormalities may be present and include asymmetrical microphthalmia, cataracts, optic nerve atrophy, coloboma, lens defects, and herniation of the orbital fat into the cranial vault [74]. Finally, it is important to note that even if severe microcephaly is the most frequent feature, normal head circumference does not exclude ZIKV infection and neonates can have severe brain abnormalities with a normal head circumference. In terms of therapeutic options there is currently no specific therapy for Zika virus [75].

**Figure 14:**
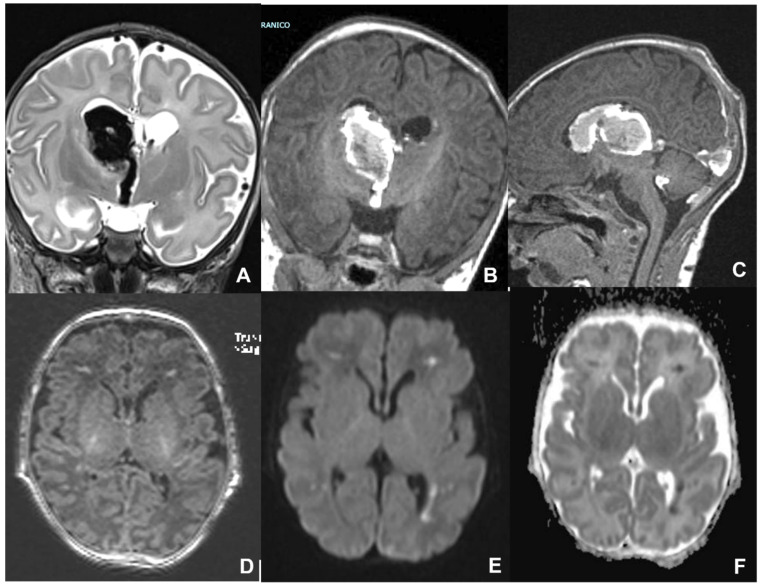
Term neonate affected by SARS-CoV2 undergoing MRI at five days after birth, showing supratentorial and infratentorial hemoventricle coexisting with subarachnoid hemorrhage on coronal T2WI-T1WI (**A**,**B**) and sagittal T1 (**C**). Term neonate affected by SARS-CoV-2 undergoing MRI at three days after birth, showing bilateral frontal hemorrhagic foci and linear subependymal hemorrhage on axial T1WI (**D**) and DWI/ADC (**E**,**F**).

The recent COVID-19 pandemic caused by SARS-CoV-2 is affecting millions of people of all ages around the world with a wide spectrum of clinical presentations ranging from asymptomatic to severe interstitial pneumonia, hyperinflammation, and death. It is still debated whether SARS-CoV-2 should be considered a “TORCH” infection [76]. Indeed, SARS-CoV-2 vertical transmission is still controversial with some studies excluding that possibility [77,78], while some recent case reports document a possible in utero transmission [79,80]. On the other hand, even without a vertical transmission, SARS-CoV-2 infection in pregnancy may cause indirect neonatal sequelae. SARS-CoV-2 can infect the placenta and compromise its function, leading, in rare cases, to fetal distress, preterm delivery, and perinatal asphyxia with possible brain damage ranging from periventricular leukomalacia, intraventricular and parenchymal hemorrhages to severe anoxic lesions [80] (Figure 14). The known prothrombotic status induced by SARS-CoV-2 infection [81] may play a crucial role in the development of severe neonatal complications, such as cerebral vein thrombosis, even without a transplacental transmission [82]. Recent literature has documented that birth during the pandemic correlated with differences in neurodevelopment at six months [83]. To date, there are no specific guidelines in terms of therapy for COVID-19 infection in newborns and, therefore, the treatment is defined case by case [84]. The World Health Organization (WHO) recommends COVID-19 vaccination for people who are pregnant, breastfeeding, trying to get pregnant now, or who might become pregnant in the future [85].

#### Parvovirus

Human parvovirus B19 (B19V) is a single-stranded DNA virus, a member of the Parvoviridae family, and is known to infect humans exclusively. In childhood, it causes the fifth disease, presenting with fever and flu-like symptoms followed by a "slapped cheek” rash [36,86]. Among the “other” group of congenital infection agents, human parvovirus B19 (B19V) represents a peculiarity, since nearly 55 to 65% of women of reproductive age have protective specific IgG antibodies due to seasonal and annual outbreaks in schools and nurseries. The remaining women have susceptibility to infection with a vertical rate of transmission of around 33%. The virus may be detected by performing a PCR test on amniocental or cordocental specimens [87].

**Figure 15:**
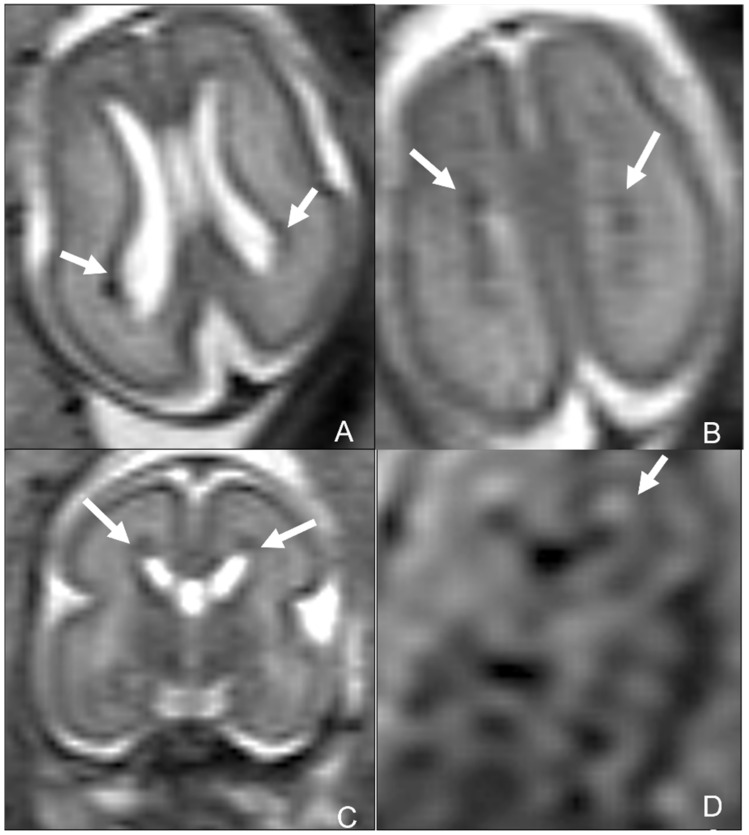
Fetus of 23 weeks of gestation age affected by parvovirus and presenting with anemia, cardiac failure, hydrops, and venous congestion. Fetal MRI shows hemorrhagic venous infarctions in deep medullary veins (white arrows in (**A**–**D**)).

Neuroimaging data of congenital infection by B19V is extremely limited. The earlier the transmission is (i.e., before the 22nd week of pregnancy), the higher the rate and the worse the complications are. Among the most frequent intra-uterine complications we account for are anemia, which is related to B19V infection of human erythroid progenitors in the bone marrow; fetal hydrops, with a higher risk if the infection occurs during the second semester of gestation; and fetal demise, with a percentage as high as 25% in the first trimester. Parvovirus may also present as a severe cerebral vasculitis leading to arterial infarction and/or parenchymal hemorrhages associated with hydrops and fetal blood transfusion. Rare cases of polymicrogyria and heterotopia have been described. The fetal US may represent the first imaging tool and shows ventricular dilatation. Fetal ecocolor-Doppler and cerebral artery systolic flow velocity measurements may assess in-utero anemia. Fetal and post-natal MRIs better depict ventricular dilatation and B19V complications as cerebral stroke showing diffusion restriction on DWI/ADC and hyperintensity on T1 following the hemorrhagic transformation of cerebral infarction or cerebral hemorrhages [70,88,89,90,91,92] (Figure 15).

Treatment should pair clinical and laboratory presentation and consist of cordocentesis transfusion, which may reverse hydrops with fetal recovery and survival. Follow-ups with fetal and post-natal MRIs are crucial to evaluate the fetus/neonate progression and suggest the administration of blood products [70].

## 3. Conclusions

Congenital infections, including TORCH group infections, are challenging infections because the clinical and radiological pictures are manifold. The different gestational period in which the woman contracts the infection affects clinical symptoms and radiological features, which can overlap even when generated by different pathogens. In most cases, early infections are related to a worse prognosis and severe neurological sequelae. To prevent long-term adverse outcomes, multidisciplinary collaboration at the time of diagnosis and long-term follow-up are fundamental. The clinician needs the support of the radiologist to correctly evaluate diagnosis, prognosis, and the duration of therapy. Long-term follow-up is essential, as many of the outcomes, such as deafness, blindness and neurodevelopmental delay, can occur later. Therefore, the repetition of neuroimaging and the constant collaboration with clinicians represent an indisputable necessity to implement preventive medicine in children exposed to infection in utero.

## Figures and Tables

**Table 1:** Main radiological features of TORCH infections.

	Calcifications	Ventricles	Cortex	White Matter	Other
**CMV**	Periventricular, punctate	Ventriculomegaly	Polymicrogyria, lissencephaly, schizencephaly	Periventriculardelayed myelination, Germinolitic cysts, Temporal cyst	Hemorrhage (rare). Cerebellum hypoplasia
**TOXOPLASMOSIS**	Extensive and most commonly in basal ganglia, thalami, cerebral cortex, and periventricular tissue	Hydrocephalus, Hydranencephaly (rare)	Microcephaly, Macrocephaly, Porencephaly	Microcephaly, Macrocephaly, Porencephaly	Aqueduct occlusion/stenosis secondary to epididymitis
**HSV**	Less common	Ventriculomegaly, hydranencephaly	Microcephaly, Porencephaly	Microcephaly, Porencephaly	Corpus callosum agenesis, microphthalmia, meningeal involvement
**RUBELLA**	Basal ganglia and periventricular	Ventriculomegaly	Polymicrogyria	Extensive multifocal white matter hyperintensity, mainly in the frontal lobes	Subependymal cysts, leucostriate vasculopathy, myelination delay, cerebellar hypoplasia
**PARVOVIRUS**	Less common	Ventricular dilatation	Ischemic and hemorrhagic strokes, Polymicrogyria (rare), heterotopia (rare)	Ischemic and hemorrhagic strokes	Cerebral vasculitis, hydrops
**SARS-COVID19**	/	Intraventricular hemorrhage	Parenchymal hemorrhage, cerebral ischemia	Periventricular leukomalacia, parenchymal hemorrhage, cerebral ischemia	Cerebral vein thrombosis
**ZIKA VIRUS**	Corticomedullary junction in frontal and parietal lobes, less frequently in the thalamus, basal ganglia, cortex, and periventricular regions	Ventriculomegaly	Microcephaly, polymicrogyria, gyral simplification, pachygyria-lissencephaly, opercular dysplasia, heterotopia	Microcephaly, delayed myelination, dysmyelination, hypoplasia of corticospinal tracts	Pseudocysts in the occipital horns, asymmetrical microphthalmia, cataracts, optic nerve atrophy, coloboma, lens defects, herniation of the orbital fat into the cranial vault, thin spinal cord

## Data Availability

Data are available from the corresponding author, AG, upon reasonable request.

## Abbreviations

CNS	central nervous system
TORCH	Toxoplasma gondii, others, rubella, cytomegalovirus, herpes
cUS	cerebral ultrasound
MR	magnetic resonance
cCMV	congenital cytomegalovirus
CMV	cytomegalovirus
WI	weighted image
GRE	gradient-echo
SWI	susceptibility-weighted imaging
CT	computed tomography
SEM	skin, eye, mouth
DWI	diffusion-weighted image
ASL	arterial spin labeling
HIV	human immunodeficiency virus
ZIKV	zika virus
SARS-CoV-2	severe acute respiratory syndrome coronavirus-2
